# Non‐differential gut microbes contribute to hypertension and its severity through co‐abundances: A multi‐regional prospective cohort study

**DOI:** 10.1002/imt2.268

**Published:** 2025-01-10

**Authors:** Lu Liu, Qianyi Zhou, Tianbao Xu, Qiufeng Deng, Yuhao Sun, Jingxiang Fu, Muxuan Chen, Xiaojiao Chen, Zhenchao Ma, Quanbin Dong, Beining Ma, Yuwen Jiao, Yan Zhou, Tingting Wu, Huayiyang Zou, Jing Shi, Yifeng Wang, Yanhui Sheng, Liming Tang, Chao Zheng, Wei Wu, Wenjun Ma, Wei Sun, Shixian Hu, Hongwei Zhou, Yan He, Xiangqing Kong, Lianmin Chen

**Affiliations:** ^1^ Department of Cardiology, The First Affiliated Hospital of Nanjing Medical University Nanjing Medical University Nanjing China; ^2^ Changzhou Medical Center, The Affiliated Changzhou No.2 People's Hospital of Nanjing Medical University Nanjing Medical University Changzhou China; ^3^ Microbiome Medicine Center, Department of Laboratory Medicine, Zhujiang Hospital Southern Medical University Guangzhou China; ^4^ Department of Cardiology, The Affiliated Kezhou People's Hospital of Nanjing Medical University Nanjing Medical University Xinjiang China; ^5^ Huzhou Central Hospital, Affiliated Huzhou Hospital Zhejiang University School of Medicine Huzhou China; ^6^ Cardiovascular Research Center, The Affiliated Suzhou Hospital of Nanjing Medical University, Suzhou Municipal Hospital, Gusu School Nanjing Medical University Suzhou China; ^7^ Department of Endocrinology, The Second Affiliated Hospital, School of Medicine Zhejiang University Hangzhou China; ^8^ Guangdong Provincial Institute of Public Health Guangdong Provincial Center for Disease Control and Prevention Guangzhou China; ^9^ Department of Public Health and Preventive Medicine, School of Medicine Jinan University Guangzhou China; ^10^ Institute of Precision Medicine, The First Affiliated Hospital Sun Yat‐sen University Guangzhou China; ^11^ Guangdong Provincial Clinical Research Center for Laboratory Medicine Guangzhou China; ^12^ State Key Laboratory of Organ Failure Research Southern Medical University Guangzhou China; ^13^ Key Laboratory of Mental Health of the Ministry of Education Guangzhou China

**Keywords:** co‐abundance, cohort study, gut microbiota, hypertension

## Abstract

Microbial dysbiosis, characterized by an imbalanced microbial community structure and function, has been linked to hypertension. While prior research has primarily focused on differential abundances, our study highlights the role of non‐differential microbes in hypertension. We propose that non‐differential microbes contribute to hypertension through their ecological interactions, as defined by co‐abundances (pairs of microbes exhibiting correlated abundance patterns). Using gut microbiome data from the Guangdong Gut Microbiome Project, which includes 2355 hypertensive and 4644 non‐hypertensive participants across 14 regions, we identified replicable hypertension‐related microbial interactions. Notably, most co‐abundances involved non‐differential microbes, which were found to correlate with both hypertension severity and hypertension‐related microbial metabolic pathways. These findings emphasize the importance of microbial interactions in hypertension pathogenesis and propose a novel perspective for microbiome‐based therapeutic strategies.

## Highlights

- Gut microbial co‐abundances differ between hypertensive and non‐hypertensive participants.
- Non‐differential gut microbes dominate hypertension‐related co‐abundances.
- The strength of gut microbial co‐abundances is associated with hypertension severity.
- Microbial interactions contribute additional variance to hypertension‐related microbial metabolic pathways.

## Graphical abstract

In this study, we examine the disruption of microbial ecological interactions, as reflected by co‐abundances, in relation to hypertension and its severity in a large‐scale, population‐based cohort from the Guangdong Gut Microbiome Project (GGMP, *n* = 6999). We characterized 581 hypertension‐related co‐abundances that are robust against comorbidities and exhibit a 68.7% replication rate in two independent cohorts. Notably, 40.3% of genera without significant differential abundance, yet they contribute to majority (68.2%) of the hypertension‐related co‐abundances. Additionally, 14.1% of these co‐abundances varied across different hypertension grades, with the strength of 21 differential co‐abundances significantly associated with hypertension severity, including key ecology niches dominated by *Comamonas* and *Ruminococcaceae*. Importantly, the interactions between microbes in these co‐abundances contribute extra variance to hypertension‐related pathways, such as tryptophan degradation, methionine, and O‐antigen biosynthesis.

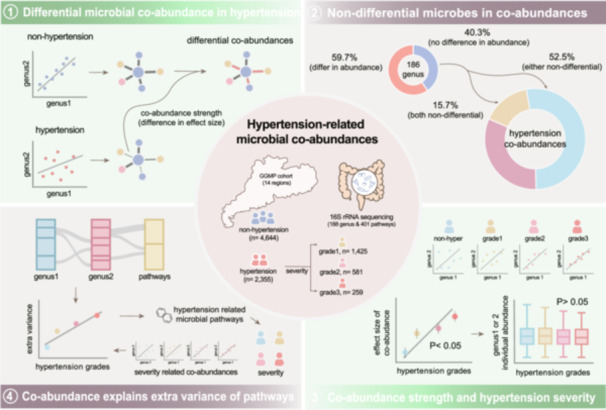

## INTRODUCTION

Hypertension is one of the most prevalent chronic cardiovascular diseases, contributing significantly to mortality rates and inducing target‐organ damage such as stroke, heart failure, chronic kidney disease, and other associated conditions [1, 2, 3]. With over 1.2 billion adults affected worldwide, hypertension poses a considerable burden on public health [4, 5]. The pathogenesis of hypertension is multifaceted, involving a complex interplay of genetic and environmental factors. In recent years, mounting evidence has underscored the intricate relationship between gut microbiota and hypertension, demonstrating their capacity to influence blood pressure through diverse mechanistic pathways [6, 7]. Previous research has elucidated dysbiosis of the gut microbiota in hypertension [8, 9, 10, 11, 12, 13, 14, 15, 16, 17, 18]. In comparison to individuals without hypertension, there is a notable reduction in microbial diversity, alongside perturbations in community structure and functionality. These investigations have been conducted extensively, spanning both animal models and human cohorts across diverse geographical regions and ethnicities [8, 13, 17]. However, the existing literature predominantly emphasizes microbial diversity, community composition, and alterations in abundance of species and pathways while overlooking the ecological interplay among microbial communities.

Indeed, the gut microbiome represents a complex ecosystem wherein microbes do not exist in isolation [19, 20, 21]. They are intricately interconnected and capable of altering community structure through mechanisms such as commensalism, competition, predation, and other forms of interaction [22, 23]. Additionally, these microorganisms interact through various metabolic activities, thereby influencing host health [24]. Inference of potential interactions between microbes by in silico method, like co‐abundance analysis, to decipher microbial interactions has gradually gained traction in disease studies by us and others, including investigations into inflammatory bowel disease [25], colorectal cancer [26], gestational diabetes [27], and nonalcoholic fatty liver disease [28]. These studies construct microbial interaction networks to identify potential key microbes associated with both health and disease states [25, 26, 27, 28, 29, 30, 31]. It is noteworthy that, as of present, the systematic exploration of ecological interactions among microbes in prospective populations with regard to hypertension, as well as their associations with hypertension severity, remains lacking.

**Figure 1:**
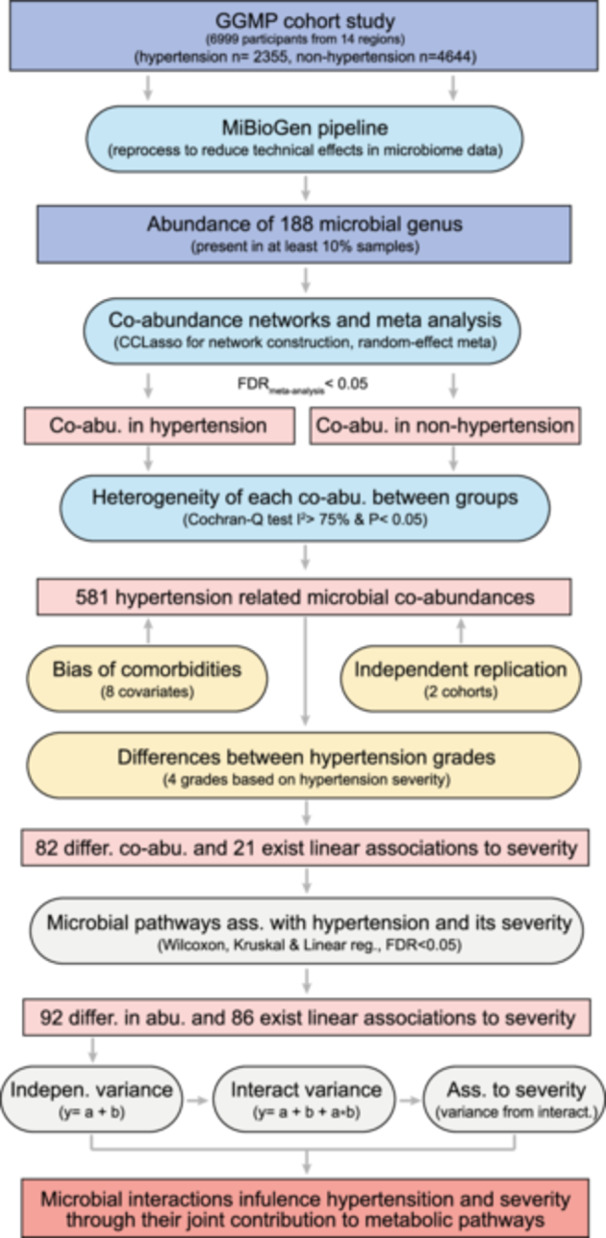
Overview of the study design and analysis workflow. This study involves a total of 2355 hypertensive and 4644 non‐hypertensive participants from 14 regions of the Guangdong Gut Microbiome Project (GGMP). For the microbial co‐abundance network inference, we included 188 bacterial genera that were present in at least 10% of the GGMP cohort. Additionally, we obtained 281 hypertension samples from The Gut Microbiome and Longevity Project (GMLP) and an Australian cohort for independent replication.

In this study, we utilized data from our Guangdong Gut Microbiome Project (GGMP) [32], a large‐scale population‐based cohort encompassing 14 regions in south of China. The cohort comprised 4644 non‐hypertensive individuals and 2355 patients diagnosed with hypertension. Through co‐abundance network analysis, we aimed to characterize microbial interactions associated with hypertension and its severity, with a focus on identifying potential key microbes shaping host‐associated microbial networks (Figure 1). Our findings revealed significant variations in microbial co‐abundances in hypertension and unveiled networks associated with hypertension severity, thereby enhancing our understanding of microbial dysbiosis in hypertension.

## RESULTS

### Inter‐microbial interactions as reflected by co‐abundances in hypertension

The dysbiosis of the gut microbiome in hypertension has been extensively characterized, with a primary focus on microbial diversity and perturbations in community structure and functionality, as evidenced by differential species and pathway abundances [8, 9, 10, 11, 12, 13, 14, 15, 16, 17]. However, there has been a notable oversight regarding the ecological interplay among microbial communities. To address this gap and investigate microbial interactions in hypertension, we leveraged the gut microbiome data of 2355 hypertensive participants (defined as having systolic blood pressure, SBP ≥ 140 or diastolic blood pressure, DBP ≥ 90 mmHg) from our Guangdong Gut Microbiome Project (GGMP) [32], and a total of 188 bacterial genus present in at least 10% of the entire participant cohort (*n* = 6999) were included for microbial interaction network construction (Figure 1).

**Figure 2:**
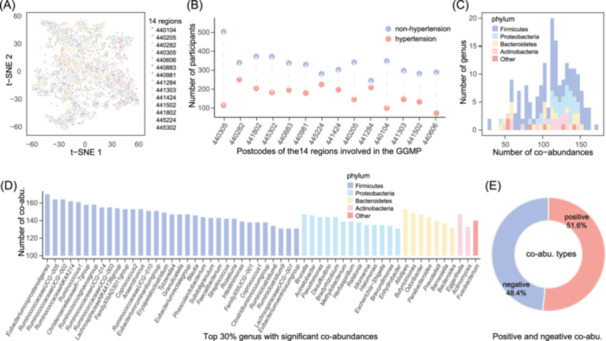
Microbial co‐abundances in hypertension. (A) Interindividual variations of gut microbial composition based on the abundance of all microbial genus detected in the GGMP cohort. Each dot represents a participant, with colors indicating participants from the 14 regions, as denoted by their respective postcodes. (B) Summary of hypertensive and non‐hypertensive participants from 14 regions. The *Y*‐axis represents the number of participants, while the *X*‐axis corresponds to the postcodes of the 14 regions in the GGMP cohort. (C) Summary of microbial genera with significant co‐abundances in hypertensive participants at false discovery rate (FDR) < 0.05. The *Y*‐axis represents the number of genera, and the *X*‐axis indicates the number of significant co‐abundances identified in hypertensive participants at FDR < 0.05 using random Meta‐analysis. Colors indicate the taxonomic classification of microbial genera at the phylum level. (D) Top 30% of microbial genera with the most significant co‐abundances in hypertensive participants. The *Y*‐axis shows the number of significant co‐abundances at FDR < 0.05, while the *X*‐axis displays the names of the microbial genera. Colors represent the taxonomic classification of microbial genera at the phylum level. (E) Summary of microbial interactions in hypertensive participants by co‐abundance directions. The pie chart summarizes the directionality of microbial co‐abundances in hypertensive participants.

As highlighted in our prior study [32], significant regional variations in gut microbiome composition exist within this cohort. To mitigate potential bias in co‐abundance network construction, we applied the MiBioGen pipeline [33] to reprocess the microbiome data using a rarefaction strategy, and nonobvious stratifications between regions were observed (Figure 2A). We then employed the CCLasso method [34] to infer the co‐abundance network separately for 14 regions (Figure 2B), which has been validated for inferring correlations among latent variables from raw compositional data through Lasso regularization, effectively mitigating overfitting due to collinearity or high dimensionality of variables [34, 35]. Subsequently, a random‐effect meta‐analysis of microbial co‐abundance networks from 14 regions was conducted based on the effect size of co‐abundance (see Methods).

Through this approach, we identified 10,454 significant microbial co‐abundances (false discovery rate (FDR) < 0.05) across 188 genera in individuals with hypertension (Table S1). By summarizing the properties of the microbial co‐abundance network in hypertension, we observed that the network encompasses microbes primarily distributed across 8 phyla, including *Firmicutes*, *Proteobacteria*, *Bacteroidetes*, *Actinobacteria*, and others (Table S2). Notably, *Firmicutes* phylum constitutes the highest participation proportion, accounting for 58% (109 out of 188) of the total genera involved and 82% (8547 out of 10,454) of co‐abundances in the network (Figure 2C). The significant co‐abundances associated with each genus range from 28 to 170, with an average of 111.2 (FDR < 0.05, Figure 2C). Interestingly, genera exhibiting the highest number of connections have been previously reported to potentially associate with hypertension, such as *Eubacterium coprostanoligenes.id.11375* [36], *Ruminococcaceae UCG‐005.id.11363*, *Ruminococcaceae UCG‐002.id.11360* [36], *Ruminococcaceae NK4A214.id.11358* [37], *Christensenellaceae R‐7.id.11283* [38], and *Ruminococcus 1. id.11373* [39] (Figure 2D). Furthermore, our analysis revealed that negative co‐abundances (interactions with a negative effect of one partner over the other) constitute 48.4% of the entire network (Figure 2E).

### Hundreds of interactions among gut microbes vary in hypertension

**Figure 3:**
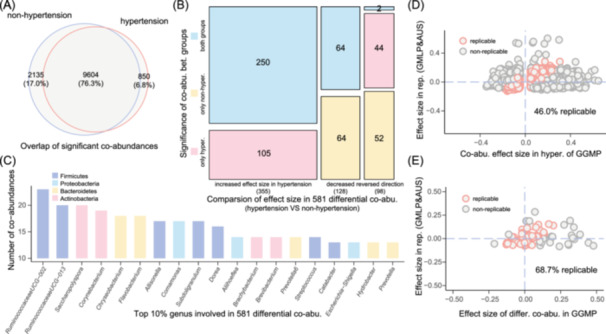
Differential microbial co‐abundances in hypertension. (A) Overlap of significant microbial co‐abundances characterized in hypertensive and non‐hypertensive participants at FDR < 0.05. (B) Summary of 581 differential microbial co‐abundances in hypertension (Cochran‐Q test, *I* ^2^ > 75% and *p* < 0.05). The *X*‐axis represents the effect size of differential co‐abundances when comparing hypertensive participants with non‐hypertensive participants. The Y*‐*axis categorizes the differential co‐abundances based on their significance in hypertensive or non‐hypertensive participants. Specifically, 355 out of 581 (61.1%) co‐abundances showed increased strength in hypertensive individuals, with 105 being significant only in hypertension (Meta‐analysis, FDR < 0.05). In contrast, 128 co‐abundances (22.0%) exhibited decreased strength, including 64 that were not significant in hypertension (Meta‐analysis, FDR > 0.05). Additionally, 98 co‐abundances (16.9%) displayed a reversal in directionality of co‐abundance strength. (C) Top 10% microbial genera with differential co‐abundances in hypertensive participants. The *Y*‐axis shows the number of differential co‐abundances identified in hypertensive participants, and the *X*‐axis displays the names of the microbial genera. Colors denote the taxonomic classification of the microbial genera at the phylum level. (D) Replication of significant co‐abundances characterized in hypertensive participants of the GGMP cohort. The *X*‐axis represents the effect size of co‐abundances identified in the GGMP cohort, while the *Y*‐axis reflects the effect size in the replication set. Each dot corresponds to a significant microbial co‐abundance identified in hypertensive participants of the GGMP cohort (Meta‐analysis, FDR < 0.05). Red dots represent replicable co‐abundances (Cochran‐Q test, *p* > 0.05), while gray dots denote non‐replicable co‐abundances (Cochran‐Q test, *p* < 0.05). (E) Replication of differential co‐abundances characterized in hypertensive participants of the GGMP cohort. The *X*‐axis indicates the effect size of differential co‐abundances identified in the GGMP cohort, and the Y‐axis corresponds to the effect size in the replication set. Each dot represents a differential microbial co‐abundance identified in hypertensive participants of the GGMP cohort. Red dots indicate replicable co‐abundances (Cochran‐Q test, *p* > 0.05), while gray dots represent non‐replicable co‐abundances (Cochran‐Q test, *p* < 0.05).

To assess the variability of microbial interactions in hypertension, we employed the same approach to construct and meta‐analyze microbial co‐abundance networks in 4644 non‐hypertensive participants from our GGMP cohort. Through this process, we identified 11,739 co‐abundances (FDR < 0.05) in non‐hypertensive participants (Table S3), with a total of 12,589 unique co‐abundances (FDR < 0.05) observed across both groups (Figure 3A). Using heterogeneity analysis, we evaluated whether the strength of each co‐abundance differed between the two groups. Our analysis revealed 581 co‐abundances with significant differences in strength (Cochran‐Q test, *I* ^2^ > 75% and *p* < 0.05, Table S4), involving 186 genera.

For hypertension‐related differential co‐abundances, we observed that 355 out of 581 (61.1%) displayed increased co‐abundance strength in hypertension compared to the non‐hypertensive group, of which 105 were only significant in hypertension (FDR < 0.05, Figure 3B). Conversely, the strength of 128 co‐abundances (22.0%) decreased, including 64 that did not reach significance in hypertension (FDR > 0.05, Figure 3B). Notably, we also observed that 98 co‐abundances (16.9%) exhibited reverse directions in co‐abundance strength (Figure 3B), indicating differences in interaction direction rather than just strength. The microbial genera with the most differential co‐abundances included *Ruminococcaceae UCG‐002.id.11360*, *Saccharopolyspora. id.757*, *Ruminococcaceae UCG‐013.id.11370*, *Corynebacterium.id.449*, and *Flavobacterium.id.1142* (Figure 3C), suggesting their central role in the network and potential key microbes in hypertension.

### Hypertension‐related microbial interactions remain unaffected by comorbidities and exhibit reproducibility

Having characterized hundreds of hypertension‐related microbial co‐abundances, we further aimed to ensure their robustness by examining potential biases associated with comorbidities and intrinsic factors, including age, sex, BMI, smoking, antibiotics, diabetes, hypercholesterolemia, and obesity (Table S5). To achieve this, we conducted a partial correlation analysis, as described in our previous work [25]. Through a heterogeneity test comparing the strength of hypertension‐related co‐abundances before and after partial correlation, we found no significant differences in microbial co‐abundances before and after correction (Cochran‐Q test *p* > 0.05, Figure S1), indicating that the identified hypertension‐related microbial co‐abundances were not affected by existing covariates.

We further assessed the reproducibility of hypertension‐related co‐abundances identified in our study across other cohorts. To accomplish this, we conducted a search for hypertension studies with available raw gut microbiome data and reprocessed 281 hypertension samples from The Gut Microbiome and Longevity Project (GMLP) [40] and an Australian cohort [41] using the same pipeline. Among the 10,454 significant co‐abundances characterized in hypertension patients in our cohort, we could evaluate 1863 co‐abundances from 83 out of 188 genera in the replication set, with 364 (19.5%) showing replication at *p* < 0.05 (CCLasso method, Table S6). The relatively low replication rate can be attributed largely to the limited sample size and overlapped genus in the replication set, as evidenced by 856 (46.0%) co‐abundances showing no significant differences in their strengths between our cohort and the replication set (Cochran‐Q test, *p* > 0.05, Table S7, Figure 3D). Besides, the environmental and genetic differences between cohorts may also contribute to this discrepancy.

Additionally, out of the 581 hypertension‐related co‐abundances, 83 could also be examined in the replication set, with 57 (68.7%) showing consistent strengths between our cohort and the replication set (Cochran‐Q test, *p* > 0.05, Table S8, Figure 3E). These findings suggest that the observed hypertension‐related microbial co‐abundances can be partly replicated in other independent cohorts.

### Nearly half of the gut microbes display non‐significance in abundance but dominate hypertension‐related interactions

**Figure 4:**
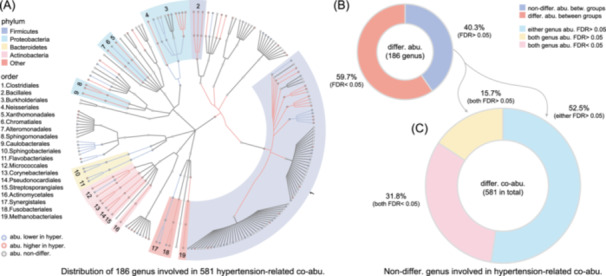
Non‐differential microbial genera involved in hypertension‐related co‐abundances. (A) Differential microbial genera at the abundance level. Red dots denote microbial genera with significantly higher abundance in hypertensive participants, while blue dots indicate those with significantly lower abundance (Wilcoxon test, FDR < 0.05). Gray dots represent microbial genera with no significant difference in abundance between the two groups (Wilcoxon test, FDR > 0.05). (B) Summary of differential and non‐differential microbial genera between hypertensive and non‐hypertensive participants. This panel contrasts the proportion of microbial genera that are differentially abundant with those that are non‐differential. (C) Summary of hypertension‐related co‐abundances based on differential and non‐differential microbial genera at the abundance level. This panel illustrates the contribution of microbial genera to hypertension‐related co‐abundances, categorized by their abundance levels. It highlights the extent to which both differential and non‐differential microbial genera participate in hypertension‐related co‐abundances, showcasing their roles in the microbial co‐abundance linked to hypertension.

Upon examination of the 186 genera involved in the 581 hypertension‐related co‐abundances (Figure 4A), 59.7% of them also exhibited significant differences in their abundance levels (Wilcoxon test, FDR < 0.05, Figure 4A,B, Table S9), and this proportion only increased to 63.4% with a looser threshold of *p* < 0.05. This suggests that the hypertension co‐abundance relationship is only partly reflected by differential microbial abundance.

Notably, 75 out of 186 genera (40.3%) did not show significance in abundance level (Wilcoxon test, FDR > 0.05, Figure 4B, Table S9), yet were involved in 396 out of 581 (68.2%) hypertension‐related co‐abundances (Figure 4C). Specifically, we observed that 91 (15.7%) hypertension‐related co‐abundances were formed by genera that did not exhibit differential abundance (Figure 4C). Our results thus indicate the microbes without significant differences in abundance (non‐differential microbes) between hypertension and non‐hypertension may also play a role in microbial dysbiosis of hypertension through their interactions reflected by co‐abundances.

### Microbial interactions vary across different grades of hypertension

**Figure 5:**
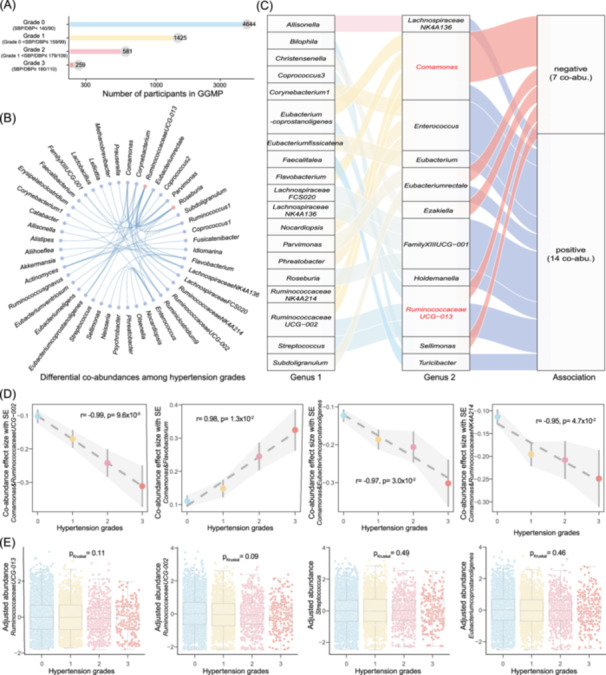
Microbial co‐abundance strength associated with hypertension severity. (A) Number of participants across different hypertension grades. The bar chart displays the distribution of participants among the various hypertension grades. The Y‐axis indicates the number of participants, and the *X*‐axis represents the distinct hypertension grades. (B) 60% of 82 differential hypertension‐related microbial co‐abundances among hypertension grades. The network plot illustrates microbial co‐abundances that show significant differences across hypertension grades (Cochran‐Q test, *I* ^2^ > 75% and *p* < 0.05). Each dot represents a microbial genus, and lines between dots indicate co‐abundance relationships between genera. Genera marked in red are those with the highest number of co‐abundances. (C) Association of 21 microbial co‐abundances with hypertension severity. The Sankey plot identifies 21 microbial co‐abundances with their effect sizes that not only vary among different hypertension grades but are also significantly associated with hypertension severity (Linear regression model, *p* < 0.05). (D) Microbial co‐abundance strength in relation to hypertension severity. The Y‐axis shows the effect size with standard errors, and the *X*‐axis indicates the different hypertension grades. Different colors denote the four hypertension grades. The dashed line represents the linear regression, with gray shading indicating the standard error. (E) Differential genera abundances among hypertension grades after covariate adjustment (Kruskal–Wallis test, FDR < 0.05). The box plot compares the normalized residuals of genera abundances among different hypertension grades after adjusting for covariates. The boxes show the median, first quartile, and third quartile (25th and 75th percentiles). The whiskers extend to the largest and smallest values within 1.5 times the interquartile range. Each dot represents a participant.

In addition to exploring differential co‐abundances related to hypertension, we further investigated whether microbial co‐abundances also exhibited differences in disease severity, specifically among different grades of hypertension. Based on clinical guidelines [42], we categorized participants in our cohorts into four types (Figure 5A), including grade 0 hypertension (SBP < 140 and DBP < 90, *n* = 4644), grade 1 hypertension (140 ≤ SBP ≤ 159, or 90 ≤ DBP ≤ 99, *n* = 1425), grade 2 hypertension (160 ≤ SBP ≤ 179, or 100 ≤ DBP ≤ 109, *n* = 581), and grade 3 hypertension (SBP ≥ 180, or DBP ≥ 110, *n* = 259), constructing microbial co‐abundance networks for each group separately (Table S10).

We then assessed if the identified 581 hypertension‐related co‐abundances also exhibited differences among the four grades. Ultimately, our analysis revealed variations in the strength of 82 hypertension‐related co‐abundance among the four grads (Cochran‐Q test, *I* ^2^ > 75% and *p* < 0.05, Table S11), indicating significant dissimilarity in microbial interactions within distinct grades of hypertension. These co‐abundances were primarily enriched in interactions related to *Ruminococcaceae UCG‐013.id.11370* and *Roseburia.id.2012* genera (Figure 5B).

### The microbial interaction strength associated with hypertension severity

Having observed heterogeneity in hypertension‐related microbial co‐abundances among different hypertension grades, we hypothesized that the strength of co‐abundances may increase or decrease with hypertension severity. To investigate this hypothesis, we examined the association between co‐abundance strength and hypertension severity for the identified 82 differential co‐abundances across different hypertension grades. Our analysis revealed that 21 of these differential co‐abundances among hypertension grades showed significant associations with hypertension severity (*p* < 0.05, Figure 5C, Table S12).

Notably, *Comamonas.id.2925* exhibited the highest number of co‐abundances associated with hypertension severity, which acts as a central player in interactions with *Eubacterium coprostanoligenes.id.11375*, *RuminococcaceaeUCG‐002.id.11360*, *Flavobacterium.id.1142*, and *Ruminococcaceae NK4A214.id.11358* (Figure 5C,D). *Comamonas.id.2925* exhibits distinctive biochemical characteristics, including the reduction of nitrate to nitrite [43, 44]. Both nitrate and nitrite are dietary sources of nitric oxide, a molecule that plays crucial roles in regulating blood pressure and vascular function [45]. Nitric oxide promotes vasodilation and improved blood flow by relaxing blood vessels, thereby aiding in blood pressure regulation [46]. The reduction of nitrate to nitrite is a microbial process primarily occurring in the gut and facilitated by complex bacterial metabolic pathways [47]. Our data thus suggest the potential for *Comamonas*‐related synergistic or antagonistic interactions with other microbes in influencing hypertension severity.

Furthermore, we observed that *RuminococcaceaeUCG‐013.id.11370* exhibits multiple co‐abundances related to hypertension severity (Figure 5C). However, all microbes involved in these co‐abundances did not show differences in their abundance levels across the four hypertension grades (Figure 5E). These findings further suggest that microbial disruptions in hypertension severity are not solely reflected at the abundance level alone. Thus, our data provide an additional layer of information that highlights microbial ecological dysbiosis in relation to hypertension severity.

### Microbial interactions may influence hypertension severity through jointly contributing to metabolic pathways

**Figure 6:**
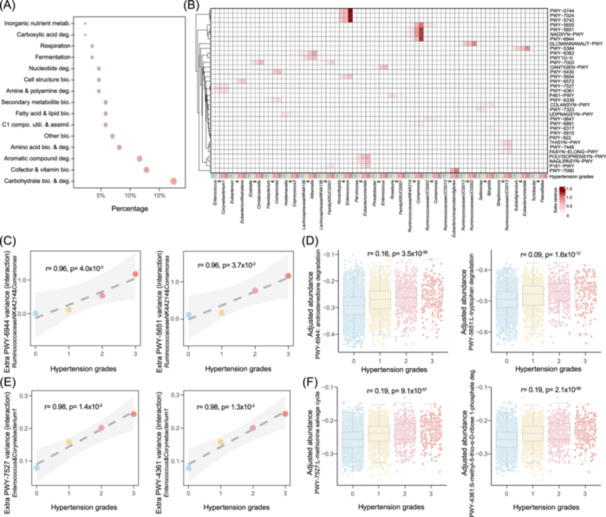
Extra metabolic pathway variances contributed by interaction terms of genera from hypertension severity‐related co‐abundances and their association with hypertension severity. (A) Functional enrichment of microbial pathways associated with hypertension severity. The *Y*‐axis lists the functional categories, while the X‐axis shows the proportion of differential pathways that fall within each metabolic category. (B) Extra variances of hypertension severity‐related pathways estimated by interaction terms among hypertension grades. The heatmap depicts the additional variances in hypertension severity‐related pathways contributed by the interaction terms between pairs of microbial genera from co‐abundances across different hypertension grades (Linear regression, *p* < 0.05). Each cell represents the extra pathway variance contributed by the interaction term between two microbial genera, with the darkness indicating the percentage of variance explained. (C) Extra variances of androstenedione and tryptophan degradation pathways positively associated with hypertension severity. The *Y*‐axis indicates the extra variance contributed by the interaction term of two microbial genera, and the *X*‐axis represents the different hypertension grades. The different colors correspond to the four hypertension grades. The dashed line denotes the linear regression, with the gray shading representing the 95% confidence interval. (D) Differential androstenedione and tryptophan degradation pathway abundances among hypertension grades after covariate adjustment (Kruskal–Wallis‐test, Spearman correlation, FDR < 0.05). The box plot compares the normalized residuals of pathway abundances for androstenedione and tryptophan degradation among different hypertension grades after adjusting for covariates. The boxes display the median, first quartile, and third quartile (25th and 75th percentiles). The whiskers extend to the largest and smallest values within 1.5 times the interquartile range. Each dot represents a participant. (E) Extra variances of two methionine biosynthesis pathways positively associated with hypertension severity. The Y‐axis indicates the extra variance contributed by the interaction term of two microbial genera, and the *X*‐axis represents the different hypertension grades. The different colors correspond to the four hypertension grades. The dashed line denotes the linear regression, with the gray shading representing the 95% confidence interval. (F) Differential methionine biosynthesis pathway abundances among hypertension grades after covariate adjustment (Kruskal–Wallis test, Spearman correlation, FDR < 0.05). The box plot compares the normalized residuals of pathway abundances for androstenedione and tryptophan degradation among different hypertension grades after adjusting for covariates. The boxes display the median, first quartile, and third quartile (25th and 75th percentiles). The whiskers extend to the largest and smallest values within 1.5 times the interquartile range. Each dot represents a participant.

To investigate the potential functional role of microbial interactions in influencing hypertension, we examined the metabolic activities of the gut microbiome by profiling their metabolic pathways. Among the pathways characterized in at least 10% of participants, we found that 92 exhibited significant differences in abundance levels between hypertensive and non‐hypertensive participants (Wilcoxon‐test, FDR < 0.05, Table S13). Of these, 86 pathways also demonstrated variations among hypertension grades (Kruskal–Wallis‐test, FDR < 0.05, Table S14), showing a linear trend with hypertension severity (FDR < 0.05, Table S14) after adjusting for the aforementioned covariates. The identified hypertension severity‐related microbial metabolic pathways encompass various categories, including short‐chain fatty acids, vitamins, amino acids, and others (Figure 6A, Table S15).

We hypothesized that microbial co‐abundances associated with hypertension severity might contribute to hypertension progression through metabolic pathways, driven by their collective interactions rather than individual contributions alone. To test this hypothesis, we introduced an interaction term between two microbes into a linear regression model to estimate the additional variance in metabolic pathways attributed to the interaction. Among the 21 identified hypertension severity‐related co‐abundances, we found that abundance variations in 84 pathways could be significantly estimated by microbes involved in hypertension severity‐related co‐abundances across the four hypertension grades (F‐test, FDR < 0.05, Table S16). Subsequently, we evaluated the additional variances estimated for a microbial pathway when the interaction term was included (Table S16). Ultimately, we characterized 40 significant associations (*p* < 0.05, Figure 6B, Table S17) between the extra variances estimated with the interaction term and hypertension severity, as reflected by the four hypertension grades.

Hypertension severity‐related co‐abundances contributed the most additional variance to pathways, including androstenedione degradation (PWY‐6944) and l‐tryptophan degradation (PWY‐5651) pathways (Figure 6B). We observed that the additional variance in both pathways, attributed to the interaction term between two genera within a co‐abundance, increased with hypertension severity (Figure 6C). Both androstenedione and l‐tryptophan have been reported to have beneficial effects on hypertension via vascular and immune regulations [48, 49]. Consistent with this, we observed that the abundance of androstenedione (PWY‐6944) and l‐tryptophan (PWY‐5651) degradation pathways increased with hypertension severity (Figure 6D).

We also found that interactions between microbes contribute more variance to l‐methionine biosynthesis pathways (PWY‐7527 and PWY‐4361, Figure 6E) as hypertension severity increases. l‐methionine is an essential amino acid for the human body and functions as a precursor to homocysteine [50]. Elevated homocysteine levels are considered a potential factor for hypertension and elevated blood pressure. Experimental and clinical evidences have shown that homocysteine is associated with hypertension [51, 52, 53, 54]. Here, we observed that the abundance of the two methionine pathways also increased with hypertension severity (Figure 6F).

Moreover, we observed that interactions between *Phreatobacter* and *Enterococcus* significantly contribute to the variance in the O‐antigen building blocks biosynthesis pathway (OANTIGEN‐PWY, Figure S2A) as hypertension severity increases. The O‐antigen, part of lipopolysaccharide, evokes specific immune responses [55, 56]. Previous studies have shown gut barrier dysfunction in hypertension patients [10, 57], allowing bacterial products like lipopolysaccharide to enter systemic circulation [58, 59], leading to systemic inflammation and endothelial cell dysfunction, which further aggravates hypertension [7, 60]. Consistent with this, we found that the abundance of the O‐antigen building blocks biosynthesis pathway increased with hypertension severity (Figure S2B).

Taken together, our analysis further suggests a potential ecological interplay between microbes in regulating hypertension severity through their joint contributions to hypertension‐related metabolic pathways.

## DISCUSSION

The human gut is colonized by a diverse community of microbes, and their disruption is closely linked to host health through various functionalities [21, 61]. Previous studies have elucidated the association between the intestinal microbiome and hypertension, primarily focusing on differential microbial diversity and abundances [8, 9, 10, 11, 12, 13, 14, 15, 16, 17]. However, the human gut represents an ecological system characterized by complex inter‐microbial interactions that remain incompletely understood. Utilizing a large‐scale, population‐based prospective cohort from 14 regions, we constructed an inter‐microbial interaction network in hypertension via co‐abundance analysis. We identified 581 co‐abundances with significant differences in strength between hypertensive and non‐hypertensive participants, independent of comorbidities, and these findings were reproducible in independent cohorts. Notably, our analysis revealed that nearly half of the microbes, although not significantly different in abundance, participate in differential co‐abundances. Furthermore, we demonstrated that microbial interactions vary across different grades of hypertension, with co‐abundance strength correlating with hypertension severity. Linking these microbial interactions to metabolic pathways, we found that microbial interactions may influence hypertension severity by collectively contributing to metabolic processes. Our study provides evidence that microbial dysbiosis in hypertension can also be reflected in alterations in microbial co‐abundance.

Over the past decade, differential abundances of microbes in health and disease have been extensively characterized and validated using both in vivo and in vitro models [8, 9, 10, 11, 12, 13, 14, 15, 16, 17]. Nevertheless, we posit that the diverse microbial communities within the human gut constitute a complex ecosystem, where microbes engage in nutrient exchange, signaling, and competition for resources or immune evasion through ecological interactions that remain incompletely understood [62, 63]. Consequently, there is growing interest in elucidating these microbial interactions to identify key microbial players in health and disease [25, 28, 30].

It is currently not possible to construct a human gut microbiota to study microbial interactions in health and disease as many microbes are not yet culturable. However, the gut microbial composition profile generated by sequencing of large human cohorts does allow us to make inferences about microbial interactions in silico by assessing their co‐abundance relationship and further comparing their differences between health and disease [25].

Using co‐abundance network analysis within our GGMP cohort, we characterized thousands of microbial interactions in hypertensive participants. Notably, many microbial genera exhibiting the highest number of co‐abundances have been previously reported to be potentially associated with hypertension, including *Eubacterium coprostanoligenes.id.11375* [36], *Ruminococcaceae UCG‐005.id.11363, Ruminococcaceae UCG‐002.id.11360* [36], *Ruminococcaceae NK4A214.id.11358* [37], *Christensenellaceae R‐7. id.11283* [38], and *Ruminococcus 1.id.11373* [39]. This suggests that microbes differentially abundant in hypertension may also play crucial roles by interacting with others.

Notably, using a heterogeneity test, we identified 581 hypertension‐related microbial co‐abundances involving 186 genera, 40.3% of which did not exhibit significant differences in abundance levels. Despite this, these genera were involved in 68.2% of the hypertension‐related co‐abundances. Specifically, we observed that 15.7% of the hypertension‐related co‐abundances were formed solely by genera that did not display differential abundance. Our findings indicate that microbes with non‐differential abundance levels may play significant roles in the microbial dysbiosis associated with hypertension through their interactions, as reflected by co‐abundance patterns. Importantly, 14.1% of the hypertension‐related co‐abundances varied across different grades of hypertension, and the strength of 21 differential co‐abundances among these grades showed significant associations with hypertension severity. These results underscore the complexity of microbial interactions in hypertension and suggest that the dysbiosis observed in this condition is influenced by a network of microbial interactions rather than by changes in individual microbial abundances alone.

To elucidate the functional role of microbial interactions in hypertension and its severity, we hypothesized that the interaction terms between pairs of microbes might contribute additional variance beyond their individual effects. Among the 21 co‐abundances associated with hypertension severity, we found that variations in the abundance of 84 metabolic pathways could be significantly predicted by the microbial pairs involved across the four grades of hypertension. Notably, we identified 40 significant associations where the additional variance explained by microbial interaction terms correlated with hypertension severity, as reflected by its four grades. These findings highlight the functional importance of microbial interactions in modulating metabolic pathways linked to hypertension progression.

These pathways include the l‐tryptophan degradation pathway, l‐methionine biosynthesis pathways, and O‐antigen building block biosynthesis pathways. For instance, l‐methionine is an essential amino acid for the human body and a precursor to homocysteine, a compound linked to hypertension [50]. Elevated homocysteine levels are considered a potential risk factor for hypertension and high blood pressure, as supported by experimental and clinical evidence [51, 52, 53, 54]. Mechanistically, homocysteine activates metalloproteinases and induces collagen synthesis, which disrupts the elastin/collagen ratio, compromising vascular elastance. Elevated homocysteine levels can lead to endothelial dysfunction, where metabolites from the hyperhomocysteinemic endothelium modify components of the underlying smooth muscle cells, contributing to the development of hypertension. Additionally, homocysteine is metabolized in the body to produce hydrogen sulfide (H2S), a potent antioxidant and vasorelaxant. However, at elevated levels, homocysteine inactivates proteins through homocysteinylation, including its endogenous metabolizing enzyme, cystathionine γ‐lyase, which reduces hydrogen sulfide production and thus exacerbates hypertension [64]. Regarding other cardiovascular threats and vascular dysfunction, methionine has been shown to upregulate eNOS/iNOS (endothelial nitric oxide synthases/inducible nitric oxide synthase, eNOS/iNOS) and MMP2/MMP9 (matrix metallopeptidase 2/matrix metallopeptidase 9, MMP2/MMP9) levels, alongside increased collagen deposition, which indicates vascular and extracellular matrix remodeling [65]. In our study, we found that the abundance of two methionine‐related pathways increased progressively with hypertension severity. These findings highlight a potential ecological interplay among gut microbes, suggesting they may regulate hypertension severity through their joint contributions to metabolic pathways associated with the condition.

In summary, this study presents an analysis of microbial networks to elucidate their relationships with hypertension in a large‐scale population‐based cohort. Our data demonstrate that dysbiosis of the gut microbial ecosystem in hypertension can be assessed not only through alterations in microbial abundance but also through changes in microbial interactions, particularly in terms of co‐abundances. We have identified potential key microbes that may play significant roles in regulating the microbial ecosystem in the context of hypertension. These findings on hypertension‐related microbial interactions deepen our understanding of the role of the microbiome in hypertension and its severity.

We acknowledge several limitations in the present study. Firstly, differences in sample sizes across groups, along with confounding factors such as diet and drug usage, may introduce bias into our results. Although we attempted to mitigate this issue by conducting heterogeneity analyses and validating our findings with independent cohorts, the potential bias from unequal sample sizes cannot be entirely ruled out. Therefore, it is essential that our findings be replicated in other independent cohorts [66, 67, 68, 69], accounting for intensive confounders, to further strengthen and validate our conclusions. Additionally, our study primarily involved the integration and analysis of data at various levels to generate plausible biological hypotheses. However, these hypotheses require further experimental validation to confirm their accuracy and relevance. Future research should focus on experimental studies to validate our findings and explore the underlying mechanisms in more detail.

## CONCLUSION

In this study, we reveal the disruption of microbial ecological interactions, as reflected by co‐abundances, associating with hypertension and its severity from a large‐scale, population‐based cohort. Beyond conventional metrics of microbial diversity and individual abundance variations, our findings demonstrate for the first time that non‐differential microbes are also important for hypertension in regulating hypertension‐related metabolic pathways through their ecological interactions. We believe that our study will offer new insights into the etiology of hypertension, as well as the design of microbiome‐based hypertension therapies.

## METHODS

### Study cohort

The Guangdong Gut Microbiome Project (GGMP) is a large population‐based cross‐sectional study conducted between 2015 and 2016 from 14 regions within Guangdong Province, China. Detailed information regarding the host metadata and sample collection for the GGMP cohort has been described previously [32]. For the purposes of this study, we included 6999 GGMP participants for whom comprehensive socio‐demographic characteristics, disease status and stool microbiome data were available. These participants consist of 55% female and 45% male, with a mean age of 53 years (standard deviation (SD) = 15). The mean body mass index (BMI) of the participants was 23.4 (SD = 3.5), and 33% were current smokers.

### Microbiome data generation and processing

For fecal sampling, participants were provided with a stool sampler, an ice pack, and detailed instructions for proper sample collection and storage. Following defecation, participants placed the fecal sample, along with the ice pack, into the provided insulated container and transported it to a nearby collection point within 24 h. The collected samples were subsequently transferred to the laboratory and stored in –80°C freezers until further processing. Total bacterial DNA was extracted using a Fecal DNA Bead Isolation Kit (Bioeasy), following the manufacturer's protocol. The V4 region of the 16S rRNA gene was amplified and sequenced using the Illumina HiSeq. 2500 platform (Beijing Genome Institute).

For taxonomic classification, we employed direct taxonomic binning instead of operational taxonomic unit (OTU) clustering, leveraging the recent MiBioGen consortium pipeline [33]. Initially, sequence quality filtering was conducted using Fastp (v0.23.2). Clean reads were then subsampled to 10,000 reads per sample, and sequences from all samples were concatenated into a single file. Taxonomic identification was performed using the RDP Classifier (v2.12) based on the SILVA database (v128). The taxonomic labels of each sequence for each sample were aggregated.

Microbial metabolic pathways were annotated using the PICRUSt2 pipeline [70], which infers gene function and pathway abundance based on OTUs derived from QIIME2 [71]. This approach allows for the functional profiling of microbial communities by associating sequence data with metabolic capabilities.

### Co‐abundance network inference

Microbial interaction networks were constructed using the CCLasso (Correlation inference for Compositional data through Least Absolute Shrinkage and Selection Operator) method [34, 35], which infers correlations among latent variables within raw compositional data. Traditional Pearson correlation analysis assumes that observed data represent absolute microbial abundances. However, microbiome data typically reflect relative abundances, which can lead to spurious results. CCLasso addresses this issue by using a least‐squares approach with a penalty term to infer correlation networks for latent variables in compositional data. An efficient alternating direction algorithm based on the augmented Lagrangian method is employed to solve the optimization problem. Comparisons demonstrate that CCLasso outperforms existing methods, such as SparCC, in recovering edges within compositional data networks. This method is also particularly effective in addressing overfitting issues associated with collinearity or high dimensionality in variable sets [34, 35]. In this study, microbial count data from 188 genera with at least 10% prevalence in the GGMP cohort were utilized for network construction using the CCLasso tool. Separate co‐abundance networks were developed for hypertensive and non‐hypertensive participants across 14 distinct regions.

To ensure robust co‐abundance inference, we employed 20 iterations and 100 bootstrap resamples per network construction. This iterative approach enhances the reliability of the inferred interactions by minimizing the influence of stochastic variations. In total, we constructed 28 interaction networks, representing distinct microbial co‐abundance patterns for hypertensive and non‐hypertensive cohorts across the studied regions.

### Meta‐analysis of co‐abundance networks from different regions

To combine microbial co‐abundance networks from 14 regions, we applied the metacor() function from the R package meta (v.6.5.0) [72]. A meta‐analysis of co‐abundance networks was performed using a random‐effects model based on the effect sizes of co‐abundance (correlation). Subsequently, the FDR was calculated using the Benjamini‐Hochberg procedure.

### Heterogeneity of microbial co‐abundances

To assess the differences in microbial co‐abundances between hypertensive and non‐hypertensive groups, we used the metagen() function from the R package meta (v.6.5.0) [72] to determine the heterogeneity of the effect size (combined co‐abundance coefficients from the meta‐analysis) for each co‐abundance between the two groups. Co‐abundances with a Cochran's Q‐test *I*² value greater than 75% and a *p*‐value less than 0.05 were considered heterogeneous [73].

### Differential microbial abundances

For the 188 microbial genera and 401 metabolic pathways with a prevalence greater than 10% in the cohort, we first applied an inverse‐rank transformation to their abundance data. Subsequently, we adjusted for eight widely recognized confounding factors unrelated to our study, including age, sex, BMI, smoking status, antibiotic use, diabetes, hypercholesterolemia, and obesity, using a linear regression model. To assess differences between hypertensive and non‐hypertensive participants, as well as among the four hypertension grades, we performed Wilcoxon rank‐sum and Kruskal‐Wallis tests, respectively. The FDR was calculated using the BH procedure.

### Influence of comorbidities on hypertension‐related microbial co‐abundances

To eliminate potential biases arising from comorbidities and other covariates on hypertension‐related microbial co‐abundances, including age, sex, BMI, smoking status, antibiotic use, diabetes, hypercholesterolemia, and obesity, we applied a partial correlation analysis as previously described [25]. The Pearson correlation between genera involved in a hypertension‐related co‐abundance and each covariate was calculated to construct a correlation matrix. This matrix was then corrected using the partial correlation function cor2pcor() from the R package corpcor (v1.6.10) [74]. Subsequently, we compared the co‐abundance coefficients before and after correction using Cochran's Q‐test. A *p*‐value greater than 0.05 was considered indicative of comparable coefficients, implying that the potential influence of covariates was successfully mitigated.

### Replication of hypertension‐related microbial co‐abundances

To replicate hypertension‐related microbial co‐abundances, we conducted a comprehensive search for hypertension cohort studies with publicly available raw gut microbiome data and associated metadata. Specifically, we obtained 281 hypertension samples from The GMLP [40] and an Australian cohort [41]. These datasets were then reprocessed using the MiBioGen consortium pipeline to ensure consistency in data processing and analysis. Subsequently, we employed the CCLasso method to infer microbial co‐abundances in the acquired datasets. Finally, we conducted Cochran's Q‐test to compare the co‐abundance strength of hypertension‐related co‐abundances across different cohorts. A *p*‐value greater than 0.05 was considered indicative of comparable coefficients, suggesting robustness and consistency in the identified hypertension‐related microbial co‐abundances between cohorts.

### Hypertension severity related microbial co‐abundances

Hypertensive participants were stratified into three grades based on their SBP and DBP (Figure 5A). Subsequently, microbial co‐abundances among different hypertension grades were inferred using the CCLasso method. Heterogeneity analysis of the 581 hypertension‐related co‐abundances across the three hypertension grades, along with the non‐hypertensive group, was conducted using the Cochran‐Q test.

To identify hypertension severity‐related microbial co‐abundances, associations between hypertension grades and co‐abundance strength were examined using linear regression. A *p*‐value less than 0.05 was considered indicative of significant associations with hypertension severity.

### Estimating the variance of hypertension severity‐related pathways with microbes involved in co‐abundances

To estimate the variance of hypertension severity‐related pathways attributed to microbes involved in co‐abundances, we employed a multi‐step approach. Initially, for identified hypertension severity‐related microbial pathways, we assessed their inter‐individual variations using two microbial genera involved in a hypertension severity‐related co‐abundance, employing a binary linear regression model: $Y=\alpha +\beta_{\mathrm{genera}1}x_{\mathrm{genera}1}+\beta_{\mathrm{genera}2}x_{\mathrm{genera}2}+\varepsilon$, where Y is the hypertension severity related microbial pathway, α is the constant term, $x_{\mathrm{genera}1}$ and $x_{\mathrm{genera}2}$ are the independent variables, $\beta_{\mathrm{genera}1}$ and $\beta_{\mathrm{genera}2}$ are the regression coefficients, ε represents the error terms. Subsequently, we investigated whether the introduction of an interaction term between the two microbes could enhance the estimation of variance in hypertension severity‐related microbial metabolic pathways ($Y=\alpha +\beta_{\mathrm{genera}1}x_{\mathrm{genera}1}+\beta_{\mathrm{genera}2}x_{\mathrm{genera}2}+\beta_{\mathrm{genera}1*\mathrm{genera}2}x_{\mathrm{genera}1*\mathrm{genera}2}+\varepsilon$, where $\beta_{\mathrm{genera}1*\mathrm{genera}2}$ is coefficient of interaction term of $x_{\mathrm{genera}1*\mathrm{genera}2}$). *p* values from F‐test in the linear regression model were recorded and adjusted for multiple tests.

Finally, we examined the association between the extra variances estimated with the interaction term and hypertension severity, as indicated by hypertension grades. A significance threshold of *p*‐value less than 0.05 was applied.

## AUTHOR CONTRIBUTIONS

**Lu Liu**: Writing—original draft; visualization; writing—review and editing; methodology; validation; formal analysis. **Qianyi Zhou**: Writing—review and editing; investigation. **Tianbao Xu**: Investigation; writing—review and editing. **Qiufeng Deng**: Visualization. **Yuhao Sun**, **Beining Ma**, and **Tingting Wu**: Investigation. **Jingxiang Fu**, **Muxuan Chen**, **Xiaojiao Chen**, **Zhenchao Ma**, **Yuwen Jiao**, **Yan Zhou**, **Huayiyang Zou**, **Jing Shi**, **Yanhui Sheng**, **Liming Tang**, **Chao Zheng**, **Wenjun Ma**, and **Wei Wu**: Supervision. **Quanbin Dong**: Data curation. **Yifeng Wang** and **Wei Sun**: Supervision. **Shixian Hu**: Supervision; writing—review and editing. **Hongwei Zhou**: Supervision; resources. **Yan He**: Supervision; resources; writing—review and editing. **Xiangqing Kong**: Supervision; resources. **Lianmin Chen**: Supervision; writing—review and editing; writing—original draft; conceptualization; methodology; project administration; resources; funding acquisition; visualization.

## CONFLICT OF INTEREST STATEMENT

The authors declare no conflicts of interest.

## ETHICS STATEMENT

The GGMP cohort study was approved by the Ethical Review Committee of the Chinese Center for Disease Control and Prevention under Approval Notice No. 201519‐A. Written informed consent was obtained from all participants.

## Supporting information


**Figure S1.** Comparison of hypertension‐related co‐abundance effect sizes before and after adjusting for potential covariates.
**Figure S2.** Interactions between Phreatobacter and Enterococcus significantly contribute to the variance in the O‐antigen building blocks biosynthesis pathway as hypertension severity increases.

**Table S1.** Microbial co‐abundances in hypertension.
**Table S2.** Microbial genus involved in the hypertensive co‐abundance network.
**Table S3.** Microbial co‐abundances in non‐hypertension.
**Table S4.** 581 microbial co‐abundances show differences in hypertension.
**Table S5.** Summary of phenotypes between the non‐hypertension and hypertension groups.
**Table S6.** Replication of microbial co‐abundances identified in hypertension at *p* < 0.05.
**Table S7.** Replication of microbial co‐abundances identified in hypertension with heterogeneity test.
**Table S8.** Replication of hypertension‐related co‐abundances.
**Table S9.** Differential abundance of genus involved in hypertension‐related co‐abundances.
**Table S10.** Microbial co‐abundances from four hypertension grades.
**Table S11.** Hypertension‐related differential co‐abundances show differences among hypertension grades.
**Table S12.** 21 hypertension severity‐related microbial co‐abundances.
**Table S13.** Differential pathway abundance between hypertension and non‐hypertension.
**Table S14.** Pathway abundance associated with hypertension grades.
**Table S15.** Metabolic categories of hypertension severity‐related microbial pathways.
**Table S16.** Pathway variance estimated by genus involved in co‐abundances with and without interaction term among different hypertension grades.
**Table S17.** Extra pathway variances estimated with interaction term associated with hypertension grades.

## Data Availability

The data that support the findings of this study are openly available in the European Nucleotide Archive at https://www.ebi.ac.uk/ena/, reference number PRJEB18535. The gut microbiome sequencing data of the Guangdong Gut Microbiome Project is deposited at the European Nucleotide Archive (https://www.ebi.ac.uk/ena/) with accession number PRJEB18535. The metadata of the GGMP cohort is available in the supplementary information of the original publication [32]. Analysis codes are available via: https://github.com/MicrobiomeCardioMetaLab/HypertensionNetwork_project. Supplementary materials (figures, tables, graphical abstract, slides, videos, Chinese translated version, and update materials) may be found in the online DOI or iMeta Science http://www.imeta.science/.
