# Supplementary material for: Non‐differential gut microbes contribute to hypertension and its severity through co‐abundances: A multi‐regional prospective cohort study
Source: Imeta. 2025 Jan 10;4(1):e268. doi: 10.1002/imt2.268 (PMC11865328; doi:10.1002/imt2.268)
Supplement: Supplementary file 1 — Figure S1. Comparison of hypertension‐related co‐abundance effect sizes before and after adjusting for potential covariates. Figure S2. Interactions between Phreatobacter and Enterococcus significantly contribute to the variance in the O‐antigen building blocks biosynthesis pathway as hypertension severity increases. [file IMT2-4-e268-s002.docx]

**Supporting information to:**

**Non-differential gut microbes contribute to hypertension and its severity through co-abundances: a multi-regional prospective cohort study**

**Running title:** Hypertension-related microbial co-abundances

Lu Liu^1,2#^, Qianyi Zhou^3#^, Tianbao Xu^4#^, Qiufeng Deng^1,2^, Yuhao Sun^1^, Jingxiang Fu^3^, Muxuan Chen^3^, Xiaojiao Chen^3^, Zhenchao Ma^5^, Quanbin Dong^1^, Beining Ma^1^, Yuwen Jiao^2^, Yan Zhou^2^, Tingting Wu^1^, Huayiyang Zou^1^, Jing Shi^1^, Yifeng Wang^1^, Yanhui Sheng^6^, Liming Tang^2^, Chao Zheng^5,7^, Wei Wu^8^, Wenjun Ma^9^, Wei Sun^1^, Shixian Hu^10^, Hongwei Zhou^3,11,12^, Yan He^3,11,12,13*^, Xiangqing Kong^1,6*^ & Lianmin Chen^1,2*^

^1^ Department of Cardiology, The First Affiliated Hospital of Nanjing Medical University, Nanjing Medical University, Nanjing 210000, China

^2^ Changzhou Medical Center, The Affiliated Changzhou No.2 People's Hospital of Nanjing Medical University, Nanjing Medical University, Changzhou 213000, China

^3^ Microbiome Medicine Center, Department of Laboratory Medicine, Zhujiang Hospital, Southern Medical University, Guangzhou 510000, China

^4^ Department of Cardiology, The Affiliated Kezhou People's Hospital of Nanjing Medical University, Nanjing Medical University, Xinjiang 845350, China

^5^ Huzhou Central Hospital, Affiliated Huzhou Hospital, Zhejiang University School of Medicine, Huzhou 313000, China

^6^ Cardiovascular Research Center, The Affiliated Suzhou Hospital of Nanjing Medical University, Suzhou Municipal Hospital, Gusu School, Nanjing Medical University, Suzhou 215000, China

^7^ Department of Endocrinology, The Second Affiliated Hospital, School of Medicine, Zhejiang University, Hangzhou 310000, China

^8^ Guangdong Provincial Institute of Public Health, Guangdong Provincial Center for Disease Control and Prevention, Guangzhou 510000, China

^9^ Department of Public Health and Preventive Medicine, School of Medicine, Jinan University, Guangzhou 510000, China

^10^ Institute of Precision Medicine, The First Affiliated Hospital, Sun Yat-sen University, Guangzhou 510000, China

^11^ Guangdong Provincial Clinical Research Center for Laboratory Medicine, Guangzhou, China

^12^ State Key Laboratory of Organ Failure Research, Southern Medical University, Guangzhou 510000, China

^13^ Key Laboratory of Mental Health of the Ministry of Education, Guangzhou 510000, China

# These authors contributed equally: Lu Liu, Qianyi Zhou and Tianbao Xu

*Correspondence: [yanhe@i.smu.edu.cn](mailto:yanhe@i.smu.edu.cn) (Yan He), [kongxq@njmu.edu.cn](mailto:kongxq@njmu.edu.cn) (Xiangqing Kong) & [lianminchen@njmu.edu.cn](mailto:lianminchen@njmu.edu.cn) (Lianmin Chen)

**Supplementary figures:**

**Figure S1.** Comparison of hypertension-related co-abundance effect sizes before and after adjusting for potential covariates. Each dot represents one co-abundance. Both the X- and Y-axes represent correlation coefficients of co-abundances.

**Figure S2.** Interactions between *Phreatobacter* and *Enterococcus* significantly contribute to the variance in the O-antigen building blocks biosynthesis pathway (OANTIGEN-PWY) as hypertension severity increases.
